# Positive effect of combined exercise on adipokines levels and pubertal signs in overweight and obese girls with central precocious puberty

**DOI:** 10.1186/s12944-021-01588-5

**Published:** 2021-11-06

**Authors:** Elnaz Shokri, Ali Heidarianpour, Zahra Razavi

**Affiliations:** 1 Bu Ali Sina University, Faculty of Sport Sciences, Hamedan, Iran; 2 Pediatric Endocrinology and Metabolism, Hamedan University of Medical Sciences, Hamedan, Iran

**Keywords:** Precocious puberty, Combined exercise, Detraining, Adiponectin, Resistin, TNF-α

## Abstract

**Background:**

The prevalence of precocious puberty is increasing. Obesity has been demonstrated to be associated with changes in the adipokine profile and incidence of early puberty in girls. This study assessed the pubertal signs, the levels of adiponectin, resistin, and tumor necrosis factor-alpha (TNF-α) after 12 weeks of combined exercise and 4 weeks of detraining in overweight and obese girls with precocious puberty.

**Methods:**

Thirty overweight and obese girls (aged 7–9) with precocious puberty, who had received Triptorelin, were randomly divided into two groups (15 exercise and 15 control). Initially, serum levels of adiponectin, resistin, TNF-α, luteinising hormone (LH), and follicle-stimulating hormone (FSH) and the signs of puberty progression (bone age, uterine length, and ovarian volume) were measured. The exercise group performed 60 min of combined (aerobic and resistance) exercise three times/week for 12 weeks. The control group did not receive any exercise. 48 h after the last training session and after 4 weeks of detraining, all research variables were measured (also in the control group). The statistical method used for data analysis was repeated measures ANOVA.

**Results:**

In the exercise group, adiponectin significantly increased and resistin significantly decreased after 12 weeks. After 4 weeks of detraining, adiponectin significantly decreased, but resistin significantly increased. TNF-α levels did not change significantly during the study. There was no significant difference in all of the factors in the control group. Throughout the 16-week study period, the rate of puberty and LH significantly decreased in both exercise and control groups, but FSH, LH/FSH and ovarian volume significantly decreased in the exercise group alone (*P*<0.05).

**Conclusions:**

Combined exercise increased adiponectin and decreased resistin and the rate of puberty. However, after 4 weeks of detraining, these effects diminished but did not disappear.

**Trial registration:**

IRCT, IRCT56471. Registered 25 may 2021 - Retrospectively registered, https://fa.irct.ir/user/profile

## Background

Puberty is characterized by the growth of reproductive organs, enlargement of secondary sexual feature, accelerated growth rate, and incidence of menarche in females, which is caused by the secretion of the Gonadotropin-releasing hormone (GnRH) [1]. If this process occurs in girls before the age of 8 and in boys before the age of 9, central precocious puberty (CPP) has occurred which is associated with increased growth rate and accelerated bone age [2]. The incidence of CPP in girls increased from 89.4 to 415.3 per 100,000 from 2008 to 2014 [3]. The results of a study by Le Moal et al. (2018) showed that national annual incidence was 2.68 per 10,000 girls under the age of 9 years [4]. Precocious puberty is assessed by estimating bone age, Tanner staging of pubic hair and breast development, Basal luteinising hormone (LH), and follicle-stimulating hormone (FSH) levels, GnRH stimulation tests, Magnetic Resonance Imaging/Computerized Tomography (MRI /CT) brain, Pelvic ultrasound, and the size of uterus and ovarian [5]. The treatment used for precocious puberty is Gonadotropin-releasing hormone analogue (GnRHa) [2].

Obesity affects the timing of puberty and is probably one of the reasons for earlier trends of pubertal age. Brix et al. (2020) found that higher childhood body mass index (BMI) was related to earlier pubertal timing in both sibling-matched analysis and cohort analysis in both sexes [6]. In the obese state, the additional growth in the adipose tissue has been shown to change the adipokine profile, thereby initiating a detrimental cascade of the metabolic disturbances [7]. Two of the most important adipokines are adiponectin and resistin. Adiponectin concentration decreases with obesity [8] and resistin secretion increases in obesity [9].

Adiponectin and resistin have many impacts within the hypothalamic gonadal axis and in puberty [10, 11]. Cytokines are soluble mediators of the immune function that also regulate several endocrine systems. Meanwhile, tumor necrosis factor-alpha (TNF-α) has been shown to be involved in the Hypothalamic-Pituitary-Adrenal axis [12].

Regular exercise promotes positive adaptation in the body and is involved in the prevention and treatment of obesity, obesity-related position, and chronic inflammation [13]. The results of a meta-analysis showed that exercise significantly increased adiponectin in obese children [14]. The results related to resistin are contradictory, however; data from Marcelino et al. [15] demonstrated a significant reduction in resistin concentration while the results of a meta-analytical study indicated that exercise did not decrease resistin levels in pediatric obesity [14]. Physical activity might reduce systemic inflammation through the decreased production of macrophage or adipocyte pro-inflammatory cytokines [16]. Interestingly, the results of Nimmo et al. (2013) indicated that combination of aerobic and resistance training probably led to more improvement in the inflammatory profile [17].

On the other hand, detraining means that training-induced psychological, anatomical, and physiological adaptations are partially or completely eliminated as a result of training diminution or training discontinuation [18].

Since most of the studies on girls with precocious puberty have focused on pharmacological treatment and there is a gap of data on the effects of using pharmacological combined with non-pharmacological measures, it is hoped that this study will, for the first time, provide a deeper insight into the effect of drug and exercise combination on overweight and obese girls with central precocious puberty. Furthermore, the effects of four weeks of detraining was investigated to contribute to the current state of knowledge. Therefore, the purpose of this study was to investigate the effect of 12 weeks of combined exercise and 4 weeks of detraining following that on adiponectin, resistin, and TNF-α levels and pubertal signs (bone age, uterine length, ovarian volume, LH, and FSH) in overweight and obese girls with CPP, who were being treated with GnRHa.

## Method

### Study design and subjects

**Fig. 1:**
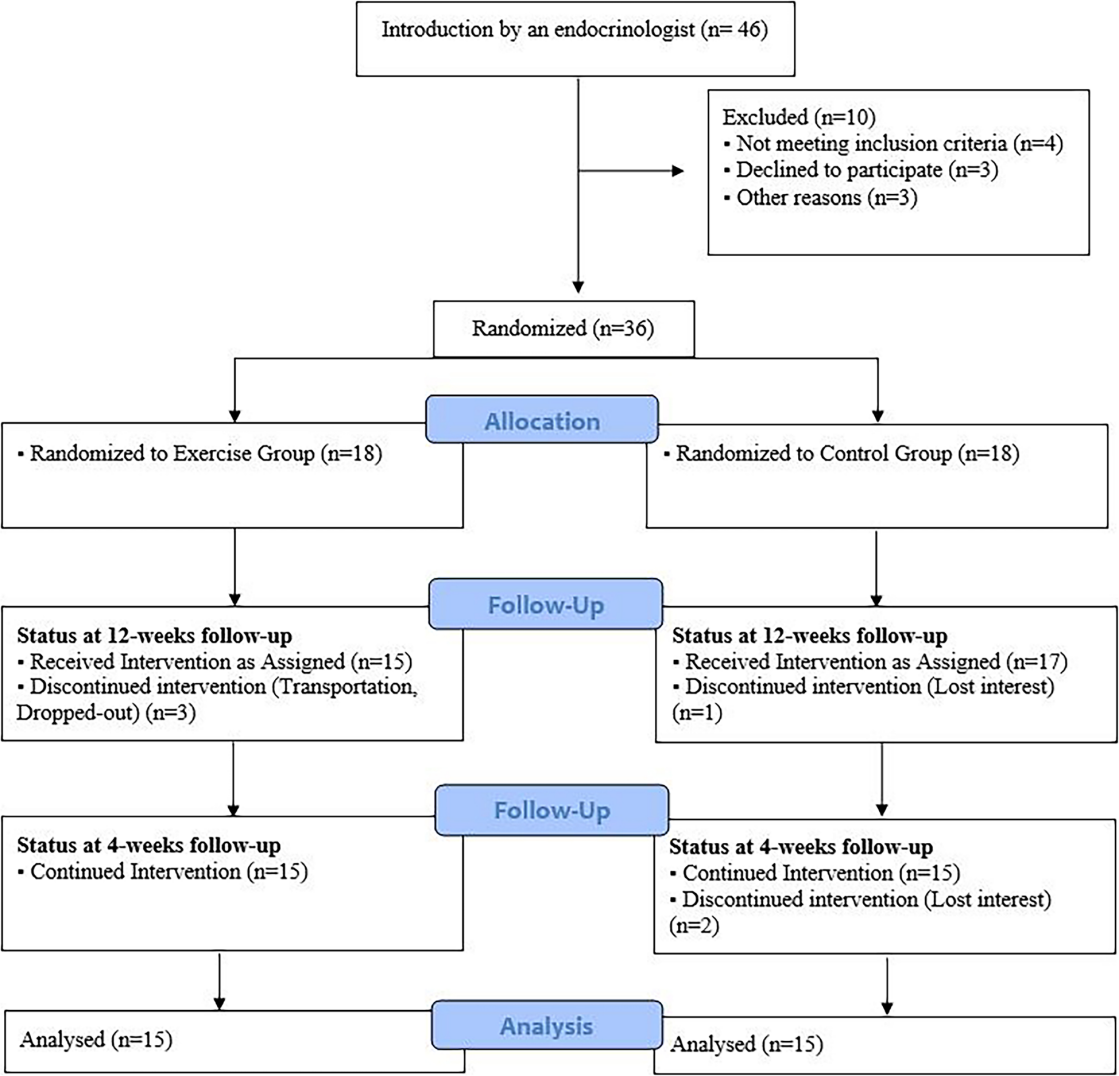
Flow diagram of the study

The statistical population of this study included all children with precocious puberty, who referred to an endocrinologist, and their precocious puberty was diagnosed based on valid criteria. Finally, Based on inclusion and exclusion criteria, out of the 46 overweight and obese girls with precocious puberty, 36 were selected and divided into two groups by the block randomization method: The exercise group (EX) performed 12 weeks of combined training (*n* = 18), and the control group (CON) did not receive any exercise (n = 18). But some left the program during the investigation and, finally, the data obtained from 30 people were analyzed (Fig. 1). Parents of all participants completed written consent form to participate in the study. The research was approved by the Research Ethics Committees of Bu Ali Sina University-Hamedan with proprietary ID IR.BASU.REC.1400.038. The trial was retrospectively registered on the IRCT (Iranian Registry of Clinical Trials).

Precocious puberty was diagnosed based on the following criteria:

- Breast growth and pubic hair: Breast growth and pubic hair growth were defined as stage 2 or higher according to Tanner [19].
- Bone age: Bone age was measured according to the method presented by Greulich and Pyle [20]; 2 years above the mean for chronological age is consistent with precocious puberty.
- Uterine and ovarian dimensions: Trans-abdominal pelvic ultrasound scans were performed with a conventional full-bladder 5 MHz scanner (SonolinePrima, Siemens); Uterine length > 3.4 cm and ovarian volume > 2.0 mL are consistent with precocious puberty [21].
- Luteinizing hormone: The hormonal evaluation included LH and FSH using a solid-phase, two-site chemiluminescent immunometric assay (Immulite2000, DPC, LosAngeles, CA, USA). An increase in levels of LH greater than 0.2 IU/L can be considered as a pubertal value [5].

All these tests and examinations were performed by an endocrinologist, a radiologist, and laboratory officials. Then, a meeting was held with the participants’ parents to brief them on the aims of the study and a general information questionnaire was distributed among them. Data from the questionnaire included the patients’ name, age, duration of disease diagnosis, duration of drug injection, type of injected drug, frequency of injection, date of the last injection, other diseases, other medicines, activity in a specific sport, training during school hours on the school campus, history of sports activity, and special diet reports. The general information questionnaire was completed by the parents during the study period. Also in this meeting, the girls’ height, weight and BMI were measured and inclusion criteria were elaborated on in more detail. The children or their legal guardian were free to leave the study whenever they wanted.

The inclusion criteria for the study included: 1. Girls with central precocious puberty; 2. BMI *above* the 85th *percentile based on the CDC* (Centers for Disease Control) *reference* [22]; 3. Being in the age range of 7 to 9; 4. On average, being diagnosed with precocious puberty for at least one year; 5. Being treated with GnRH analog (Triptorelin) 80 mg/kg (max: 3.75 mg) intramuscular every 28 days for an average of one year.

The exclusion criteria in the study included: 1. Having another illness; 2. Taking another medicine; 3. Having a special diet; 4. Being active in another sport

### Measuring the basic indicators

Height and weight of the children were measured by the children’s stadiometer (Seca 216, Germany, Hamburg) and digital weighing-scales (Beurer GS20, Germany, Ulm) respectively. Resting heart rate (RHR) was measured using a POLAR watch fastened to the wrist and measured by sensors. BMI was calculated by dividing weight by height squared (kg/m^2^) and was analyzed based on the CDC. The LMS method was used to calculate BMI Standard Deviation Score (SDS) [23]. Based on BMI, age and sex the fat mass index (FMI) and body fat percentage (% BF) were calculated using the Cortés-Castell et al.’s formula. This formula has been validated for overweight and obese children [24]. Systolic blood pressure (SBP) and Diastolic blood pressure (DBP) were measured with a Barometer (Beurer BC32 Germany, Ulm) fully automatic via evaluating the pulse on the wrist. Participants were sited during the blood pressure on a chair motionless. Measurements were repeated twice to reduce error. Peak oxygen uptake (Vo_2_peak) was measured by a 6-min walk test (6MWT) according to ATS rules. The test was conducted in a flat 30 m long corridor, where participants were instructed to walk as fast as they could without running for 6 min while listening to standardized encouragement statements [25]. This test has already been administered to and validated on children [26]. BMI, systolic and diastolic blood pressure, vo_2_peak, uterine length, and ovarian volume were measured before and after exercise and also after the detraining period in the exercise group. All of the above were also measured in the control group on all three occasions. To evaluate the effect of combined exercise, we assessed bone age after 12 weeks, but considering the fact that bone maturation should be measured yearly [27], we gave up evaluating bone age after 4 weeks of detraining because of ethical issues.

### Measuring blood samples

After measuring anthropometric indices and other primary specifications, to measure biochemical variables 24 h before the training program, blood samples were obtained from all participants. For assessment of adiponectin and resistin serum levels, commercial kits were used (EASTBIOPHARM, China, Hangzhou) with a 0.023 ng/ml and 10.23 ng/ml degree of sensitivity, respectively, and ELISA method. To measure TNF-α serum levels, commercial kits were used (BOSTER, Canada, Pleasanton) with a 1 pg/ml degree of sensitivity and ELISA method. Commercial kits were also used to measure the lipid profile (Man, Iran under license ELTTechGroup of France, Tehran). Serum levels of total cholesterol (TC) and triglycerides (TG) were measured by enzymatic procedures [28, 29]. The estimation of high-density lipoprotein (HDL) was performed using the method described by Burstein et al. [30] while the method used by Assman et al. [31] was adopted in determining low-density lipoprotein (LDL). LH and FSH were measured by electrochemiluminescence immunoassay (DxI800 automated chemiluminescence assay and commercial kit; Beckman Coulter, Inc., CA, USA) with sensitivity of 0.2 IU/L. To remove the effect of the last training session, 48 h after the last exercise session, blood samples were collected from the patients in the exercise group to evaluate the effect of the exercise program on the mentioned biochemical indices. 4 weeks after the end of the exercise, the third blood sampling was performed. The reason for choosing 4 weeks of detraining was that the results indicated that although 2 weeks of detraining is not long enough to completely eliminate the beneficial effects of regular exercise, continued detraining may lead to damaging effects [32]. All the variables were measured in the control group on all three occasions. Blood samples were collected at 8:30 to 9:00 a.m. in all three stages and from both control and exercise groups after a 12-h fast (no food or drink, except water) and at the same time.

### Intervention

The 3-month intervention in the exercise group involved a physical activity program with 3 60-min sessions/week without any dietary intervention [33]. Exercise sessions were controlled by two experienced physical education teachers and consisted of 30 min of aerobic exercise (fast walking, running, ball games) at a heart rate corresponding to 55 to 65% of individual maximal cardio-respiratory fitness (based on baseline maximal oxygen consumption [VO2max] measures by 6MWT), followed by 20 min of strengthening exercises and 10 min of stretching and cool-down. Children wore a heart rate monitor (Polar S610, Kempele, Finland) during each training session, and watch alarms warned them if the heart rate was too low (55% VO_2_max) or too high (65%VO_2_max). The aerobic period was followed by strengthening exercises of the arms, legs, and trunk (2 to 3 series of 10 to 15 repetitions), with the resistance being provided by the child’s body weight and elastic bands [33]. After the 3-month intervention, the researchers asked the participants not to participate in any exercise programs for 4 weeks. The control group did not receive any exercise in the entire 16 weeks and the researchers asked them not to participate in any exercise activities. The researchers asked both control and exercise groups to complete Physical Activity Questionnaire for Older Children (PAQ-C) to monitor their activity. This questionnaire has already been validated on Iranian children [34]. Using this questionnaire, the score of 1–1.9 was categorized as “low” and the scores between 2 and 5 as “high” physical activity [35]. If participants’ scores were in the range of 2 to 5, they would be excluded from the study. It is worth noting that the exercise group recorded activities other than the exercise program performed in the study.

### Statistical analysis

The Kolmogorov-Smirnov test was used to specify normal distribution of the data. The analysis of variance (ANOVA) with repeated measures and Bonferroni post hoc test were used to compare the difference between and within the groups. Changes (Δ1) were calculated as differences between the baseline and post-training stage (after 12 weeks). Changes (Δ2) were calculated as differences following 12 weeks and 16 weeks. The Pearson correlation coefficient was analyzed to examine the relationship between the parameter changes. The collected data were analyzed using the SPSS 20 software. The results are expressed as mean ± the standard deviation (SD). Differences were assessed statistically at *P* < 0.05.

## Results

### Analysis of questionnaires

Table 1 shows the results of the general information questionnaire and physical activity questionnaire. At the beginning of the study, mean time of onset of puberty signs in both control and exercise group was approximately 1 year. The average number of times of Triptorelin injection in both groups was 12. The results of physical activity questionnaire showed that the control group did little physical activity throughout the study period and the exercise group did not perform high physical activity except for the training protocol of the present study.

**Table 1:** The results of the general information and physical activity questionnaire

Parameters	Group	baseline		12 weeks		16 weeks	
		Mean	SD	Mean	SD	Mean	SD
Time of onset of pubertal sign (Month)	Ex	11.93	2.21	14.93	2.21	15.93	2.21
	Con	11.8	2.11	14.8	2.11	15.8	2.11
Number of Triptorelin injections	Ex	12.3	2.69	15.3	2.69	16.3	2.69
	Con	12.2	2.45	15.2	2.45	16.2	2.45
Physical activity questionnaire score	Ex	1.41	0.45	1.27	0.39	1.33	0.41
	Con	1.3	0.55	1.32	0.55	1.34	0.56

Data presented are the mean and standard deviation (SD). (Ex: Exercise group *n* = 15, Con: Control group n = 15**)**

### Analysis of physiological and anthropometric characteristics

The individual physiological and anthropometric characteristics of the participants in three stages (Baseline, 12 weeks, and 16 weeks) are presented in Table 2. Data distribution was normal. As shown in Table 2, there is no significant difference between the control and exercise groups in any of the parameters in the baseline stage. Additionally, Table 2 shows that in the exercise group, weight, BMI, BMI SDS, FMI, % BF, TC, LDL, TG, and SBP significantly decreased after 12 weeks of combined exercise (*P =* 0.001, 0.01, 0.024, 0.041, 0.04, 0.039, 0.01, 0.031, and 0.019, respectively), but HDL, 6MWT, and Vo_2_peak significantly increased after 12 weeks of combined exercise (*P* = 0.028, 0.01, and 0.036,respectively). After 4 weeks of detraining, body weight, BMI, FMI, % BF, TC, LDL, and TG significantly increased compared to 12 weeks of training (*P* = 0.032, 0.001, 0.014, 0.021, 0.035, 0.011, and 0.02, respectively) but the levels of these parameters are still significantly higher compared to the baseline stage. Also, Table 2 reveals that HDL, SBP, 6MWT, and Vo_2_peak significantly decreased after 4 weeks of detraining (*P* = 0.025, 0.017, 0.01, and 0.001, respectively). As presented in Table 2, in the control group, none of the parameters significantly changed. All the physiological and anthropometric characteristics (except RHR and DBP) were significantly different between the exercise and control group (see Table 2).

**Table 2:** Subjects’ physiological and anthropometric characteristics in three stages (Baseline, 12 weeks, 16 weeks) and two groups (Exercise and Control)

Parameters	Group	Baseline		12 weeks		16 weeks		Observed power
		Mean	SD	Mean	SD	Mean	SD	
Age (year)	Ex	8.42	0.75	8.67	0.83	8.9	0.85	–
	Con	8.28	0.64	8.53	0.66	8.61	0.71	–
Height (cm)	Ex	134.5	9.2	134.9	6.71	135	5.36	0.89
	Con	134.2	8.31	134.5	9.56	135.1	7.88	0.89
Weight (kg)	Ex	40.51	1.98	38.25^a c^	2.51	38.94^a b c^	2.33	1
	Con	39.93	1.97	40.05	1.9	40.09	1.91	0.87
BMI (kg/m^2^)	Ex	21.76	1.53	20.47^a c^	1.56	20.78 ^a b c^	1.61	1
	Con	21.75	1.71	21.76	1.72	21.76	1.72	0.55
BMI SDS	Ex	1.71	0.14	1.18 ^a c^	0.1	1.43 ^a b c^	0.13	0.97
	Con	1.72	0.23	1.75	0.24	1.77	0.18	0.94
FMI (kg/m^2^)	Ex	9.54	2.49	8.67	2.21^a c^	8.86 ^a b c^	2.25	1
	Con	9.42	2.33	9.49	2.44	9.53	2.47	0.88
BF (%)	Ex	36.57	9.7	34.20	8.5 ^a c^	34.79 ^a b c^	8.56	1
	Con	36.29	9.59	36.89	9.68	36.92	9.69	0.89
TC (mg/dl)	Ex	195.8	5.23	168.1^a c^	4.71	171.58 ^a b c^	5.39	1
	Con	196.3	6.34	193	6.41	193.8	7.38	0.88
LDL (mg/dl)	Ex	124	5.71	98.57^a c^	3.5	100 ^a b c^	4.25	0.93
	Con	124.7	6.01	123.4	5.55	123.03	6.1	0.91
HDL (mg/dl)	Ex	39	2.21	48.5^a c^	2.1	47.29 ^a b c^	3.51	0.85
	Con	39	2.54	40.2	3.59	40.55	3.92	0.77
TG (mg/dl)	Ex	83.5	3.33	72^a c^	4.2	77.33 ^a b c^	5.07	1
	Con	84	4.58	84.6	5.78	85.11	6.23	1
SBP (mmHg)	Ex	114.5	7.28	102.19^a c^	5.9	107 ^b c^	9.14	1
	Con	114.8	10.59	112.5	10.28	116.2	10.73	0.58
DBP (mmHg)	Ex	71.7	0.93	69.5	0.91	70.66	1.01	0.64
	Con	75.9	1.27	74	1.9	75	1.25	0.68
RHR	Ex	84.35	8.79	82.28	8.49	83.27	7.93	0.94
	Con	85.96	7.22	84.39	7.57	84.2	6.71	0.92
6MWT (m)	Ex	570	43	696^a c^	49	605^b^	48.58	1
	Con	581	45	599	48.1	575	47.99	0.84
Vo2peak (ml/kg/min)	Ex	37.7	4.36	40.21^a c^	5.11	38.68^b^	6.27	1
	Con	37	5.99	38.06	6.01	38.05	6.81	0.93

Data presented include the mean and standard deviation (SD). a: significantly different from baseline, b: significantly different from 12 weeks, c: significantly different from the control group. *P*<0.05 (Ex: Exercise group n = 15, Con: Control group n = 15, BMI: Body Mass Index, SDS: Standard Deviation Score, FMI: Fat Mass Index, BF: Body Fat, TC: Total Cholesterol, LDL: Low-Density Lipoprotein, HDL: High-Density Lipoprotein, TG: Triglycerides, SBP: Systolic Blood Pressure, DBP: Diastolic Blood Pressure, RHR: Resting Heart Rate, 6MWT: 6-Minute Walk Test,Vo2 peak: Peak oxygen uptake)

### Correlation analysis

Table 3 shows the Pearson correlation between parameter changes in the baseline stage and after 12 weeks of combined exercise. As shown in Table 3, changes in adiponectin, resistin, and LH after 12 weeks of combined training were correlated significantly with changes in weight and BMI. In addition, changes in some lipid profile indices were significantly correlated with changes in adiponectin, resistin, and LH. Table 4 shows the Pearson correlation between parameter changes in 12 weeks and after 4 weeks of detraining and shows that changes in adiponectin and resistin after 4 weeks of training are correlated significantly with changes in weight and BMI.

**Table 3:** Correlation between parameter changes in the baseline stage and after 12 weeks combined exercise

Parameters	Δ_**1**_ Adiponectin (μg/ml)		Δ_**1**_ Resistin (ng/ml)		Δ_**1**_ TNF-α (pg/ml)		Δ_**1**_ LH (IU/L)		Δ_**1**_ FSH (IU/L)	
	r	*P*	r	*P*	r	*P*	r	*P*	r	*P*
Δ_1_ Weight	−0.247	**0.001**	0.137	**0.023**	0.072	0.737	0.185	**0.034**	0.034	0.885
Δ_1_ BMI	−0.365	**0.016**	0.278	0.0001 *	0.171	0.067	0.145	**0.021**	0.164	0.225
Δ_1_ FMI (kg/m^2^)	−0.041	0/204	0.157	**0.012**	0.084	0.912	0.088	0.49	0.264	0.867
Δ_1_ BF (%)	−0.115	0.39	0.119	0.477	0.105	0.48	0.046	0.554	0.154	0.325
Δ_1_ TC (mg/dl)	−0.074	0.582	0.068	0.17	0.077	0.086	0.126	0.471	0.051	0.743
Δ_1_ LDL (mg/dl)	−0.355	0.04 *	0.167	0.183	−0.073	0.443	0.099	0.003 *	0.142	0.603
Δ_1_ HDL (mg/dl)	0.323	0.027 *	−0.034	0.686	−0.106	0.675	0.175	0.084	0.064	0.508
Δ_1_ TG (mg/dl)	−0.069	0.709	0.126	0.001 *	0.091	0.308	0.239	0.01*	0.152	0.037*

Δ_1_: Changes between the baseline and 12 weeks, BMI: Body Mass Index, FMI: Fat Mass Index, BF: Body Fat, TC: Total Cholesterol, LDL: Low-Density Lipoprotein, HDL: High-Density Lipoprotein, TG: Triglycerides. *: Significant correlation

**Table 4:** Correlation between parameter changes in 12 weeks and after 4 weeks detraining (16 weeks)

Parameters	Δ_**2**_ Adiponectin (μg/ml)		Δ_**2**_ Resistin (ng/ml)		Δ_**2**_ TNF-α (pg/ml)		Δ_**2**_ LH (IU/L)		Δ_**2**_ FSH (IU/L)	
	r	*P*	r	*P*	r	*P*	r	*P*	r	*P*
Δ_2_ Weight	−0.211	**0.022**	0.098	**0.01**	0.061	0.445	0.066	0.491	0.026	0.649
Δ_2_ BMI	−0.263	**0.001**	0.188	0.01 *	0.147	0.307	0.091	0.472	0.046	0.37
Δ_2_ FMI (kg/m^2^)	−0.044	0.872	0.106	0.431	0.054	0.197	0.024	0.519	0.025	0.235
Δ_2_ BF (%)	−0.108	0.464	0.033	0.909	0.031	0.706	0.009	0.438	0.016	0.247
Δ_2_ TC (mg/dl)	−0.06	0.332	0.025	0.762	0.259	0.292	0.068	0.556	0.007	0.606
Δ_2_ LDL (mg/dl)	−0.067	0.044 *	0.084	0.2	−0.022	0.881	0.099	0.003	0.035	0.58
Δ_2_ HDL (mg/dl)	0.184	0.01 *	−0.036	0.718	−0.048	0.575	0.083	0.66	0.028	0.442
Δ_2_ TG (mg/dl)	−0.05	0.439	0.039	0.041	0.026	0.703	0.048	0.217	0.077	0.115

Δ_2_: Changes between 12 weeks and 16 weeks, BMI: Body Mass Index, FMI: Fat Mass Index, BF: Body Fat, TC: Total Cholesterol, LDL: Low-Density Lipoprotein, HDL: High-Density Lipoprotein, TG: Triglycerides. *: Significant correlation

### Evaluation of pubertal signs

**Fig. 2:**
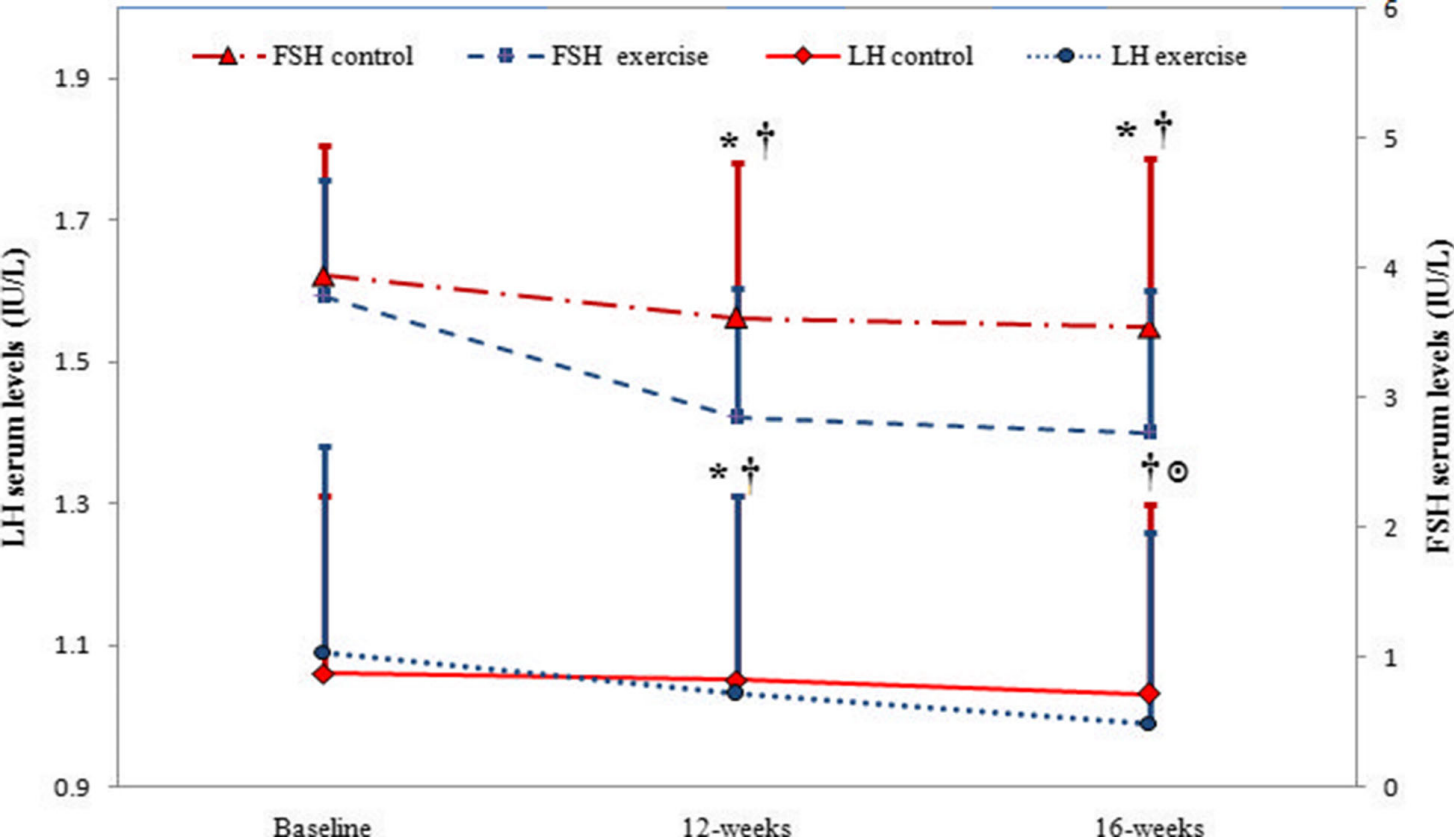
Changes in LH and FSH from the baseline to the end of 16 weeks of study in control and exercise groups (LH: Luteinising Hormone, FSH: Follicle-Stimulating Hormone). Data are reported as Mean ± SEM *: significantly different from baseline in exercise group, ๏: significantly different from baseline in control group, †:significantly different from the control group. *P*<0.05

Figure 2 displays the changes in LH and FSH levels during the study period in both control and exercise groups. The diagrams in Fig. 2 reveal that LH and FSH decreased significantly after 12 weeks of combined exercise and 4 weeks of detraining in the exercise group while in the control group, LH decreased significantly after 16 weeks of treatment. Ironically, FSH levels, did not significantly decrease in the control group even after 16 weeks (Fig. 2). Figure 2 also indicates that after 12 weeks, both LH and FSH levels are lower in the exercise group than in the control group. After 16 weeks in the exercise group, even after stopping training, the LH and FSH levels were still lower compared to the control group (Fig. 2).

**Fig. 3:**
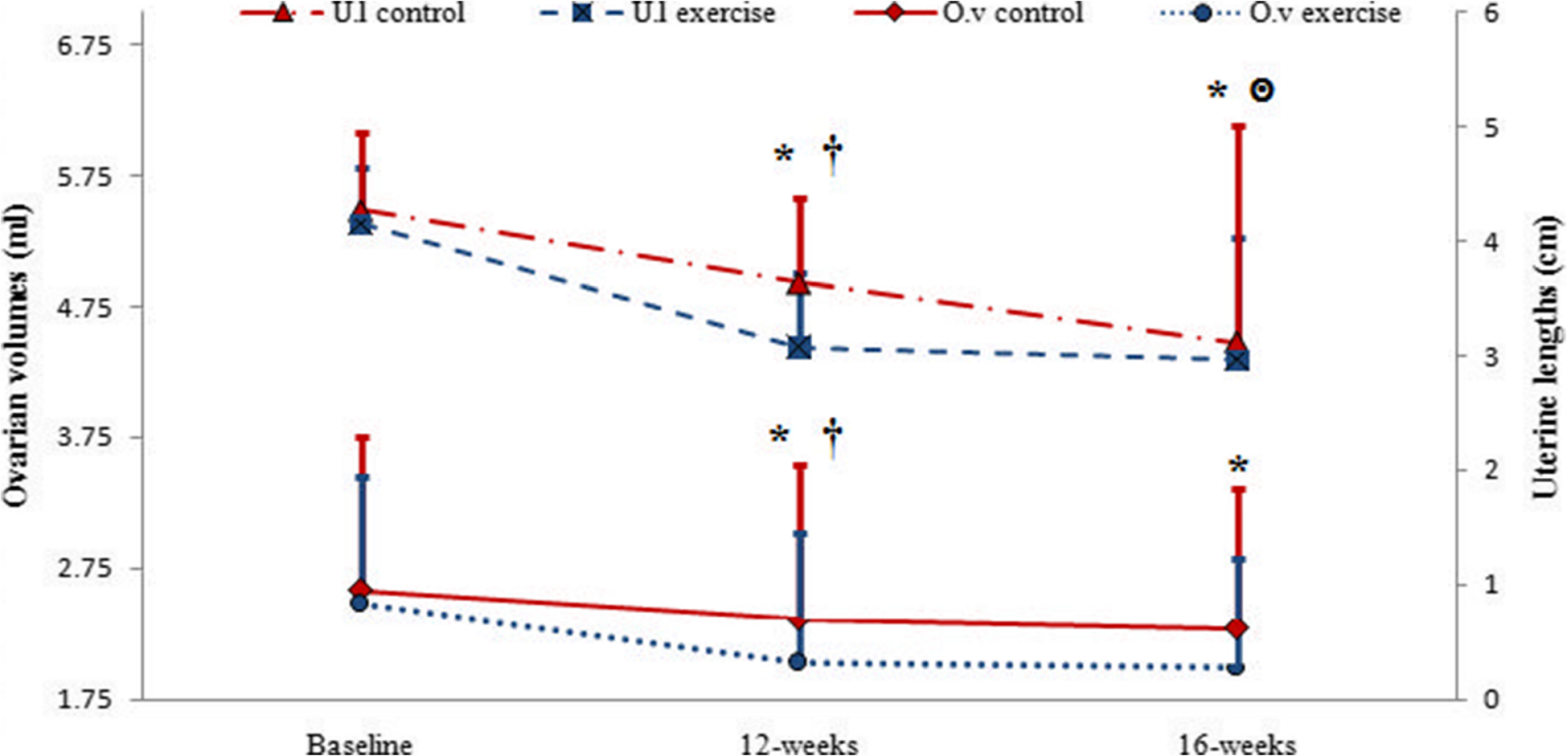
Changes in uterine length and ovarian volume from the baseline to the end of 16 weeks of study in control and exercise groups (U.l: uterine length, O.v: ovarian volume). Data are reported as Mean ± SEM *: significantly different from baseline in exercise group, ๏: significantly different from baseline in control group, †: significantly different from the control group. *P*<0.05

Changes in uterine length and ovarian volume during the study are displayed in Fig. 3. It can be deduced from the information in Fig. 3 that uterine and ovarian size decreased significantly after 12 weeks of combined exercise and 4 weeks of detraining in the exercise group while in the control group, uterine length decreased significantly after 16 weeks of treatment. Ironically, ovarian volume did not significantly decrease in the control group even after 16 weeks (Fig. 3). Also Fig. 3 indicates that after 12 weeks, both uterine length and ovarian volume are lower in the exercise group than in the control group. After 16 weeks in the exercise group, even after training was stopped, the uterine length and ovarian volume were still lower in comparison to the control group (Fig. 3).

**Fig. 4:**
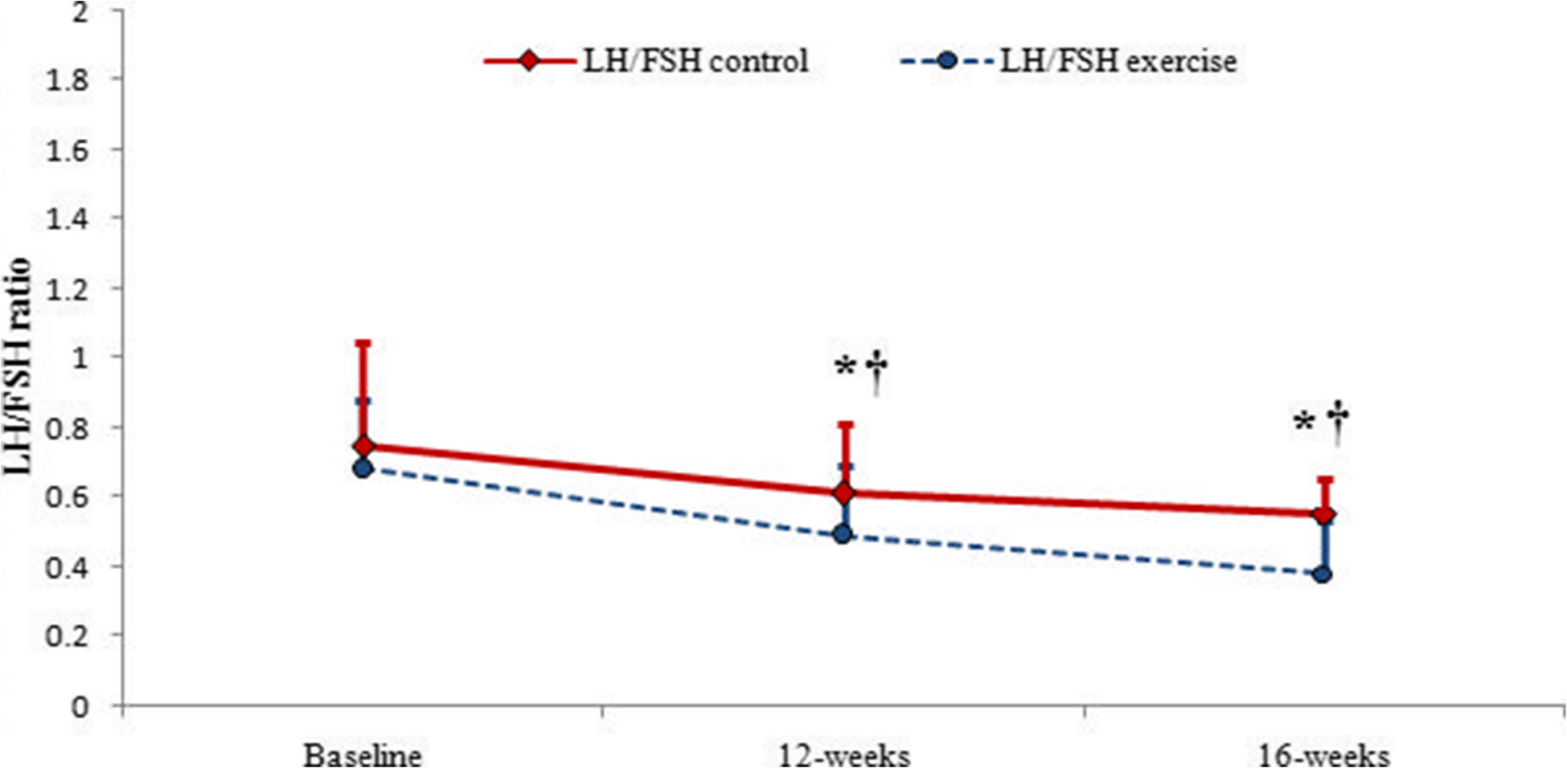
Changes in LH to FSH ratio from the baseline to the end of 16 weeks of study in control and exercise groups (LH: Luteinising Hormone, FSH: Follicle-Stimulating Hormone). Data are reported as Mean ± SEM *: significantly different from baseline in exercise group, †: significantly different from the control group. *P*<0.05

Figure 4 demonstrates the changes in the LH to FSH ratio in the two groups of control and exercise in three stages of besline, 12 weeks and 16 weeks. Figure 4 demonstrates that LH/FSH ratio decreased significantly after 12 weeks of combined exercise and 4 weeks of detraining in the exercise group, but LH/FSH did not significantly decrease in the control group even after 16 weeks (Fig. 4).

**Fig. 5:**
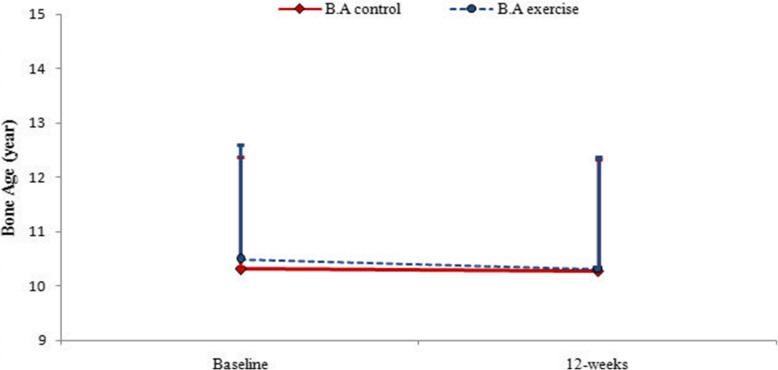
Changes in Bone Age from the baseline to the end of 16 weeks of study in control and exercise groups (B.A: Bone Age). Data are reported as Mean ± SEM

Figure 5 indicates that bone age did not change significantly after 12 weeks in any of the control and exercise groups. In exercise group and baseline stage, the difference between chronological age and bone age was 2.07 ± 0.83 years. 12 weeks after exercise, this difference reached 1.64 ± 0.63 years. In the control group, the difference between bone age and chronological age in baseline was 2.03 ± 1.24 years. At the end of 12 weeks of treatment, this difference was 1.75 ± 0.68 years (Fig. 5).

### Analysis of adipokines and cytokines

**Fig. 6:**
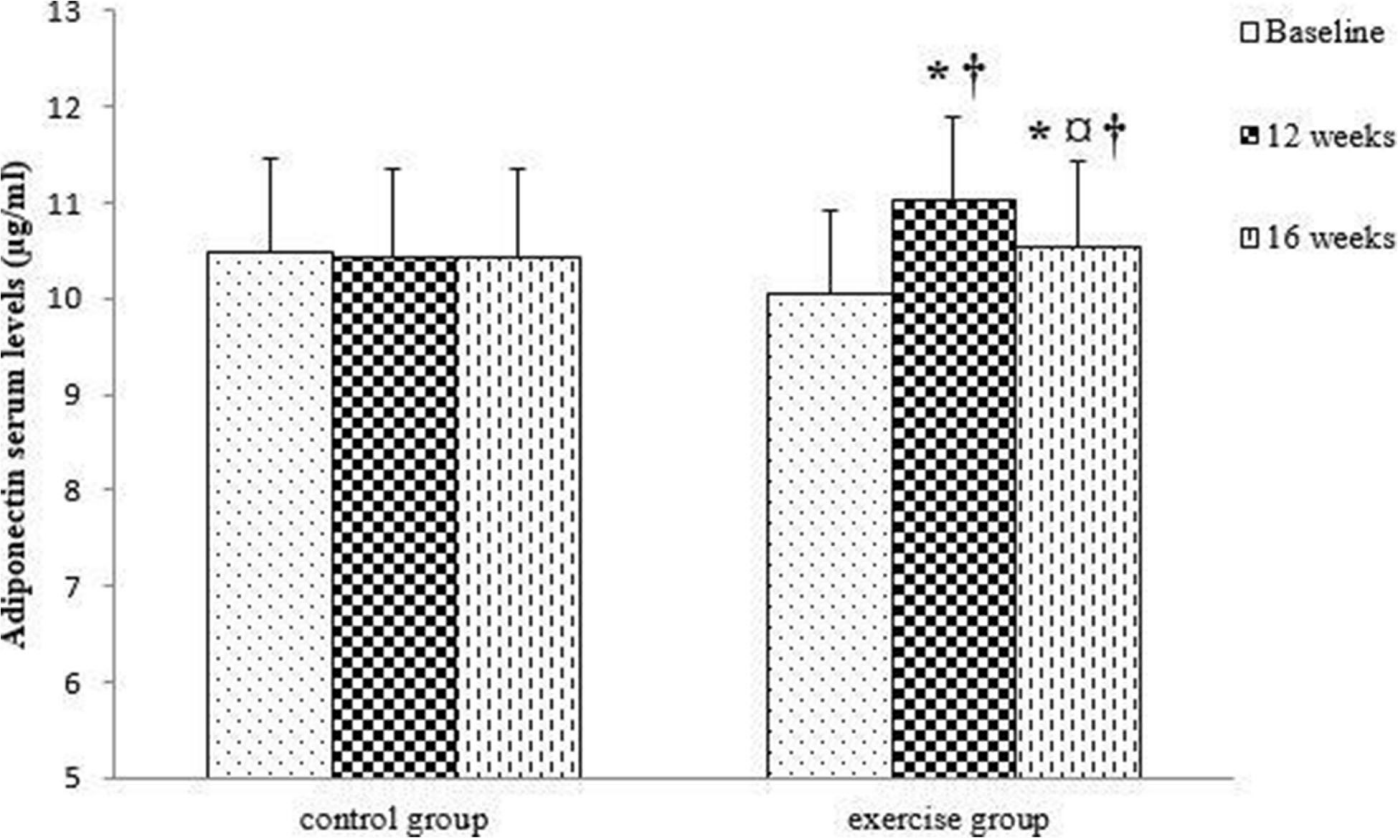
Comparison of serum levels of adiponectin in the two groups (control and exercise) and in three times (baseline, 12 weeks, 16 weeks). Data are reported as Mean ± SEM *: significantly different from baseline,¤: significantly different from 12 weeks, †: significantly different from the control group. p<0.05

The results of repeated measures ANOVA and Bonferroni post hoc test showed that adiponectin levels increased significantly after 12 weeks of combined exercise and decreased significantly after 4 weeks of detraining. However, adiponectin levels were still significantly higher compared to the baseline levels. In the control group, adiponectin levels did not change significantly during 16 weeks of study. There was a significant difference between the control and exercise groups after 12 weeks and 16 weeks (Fig. 6).

**Fig. 7:**
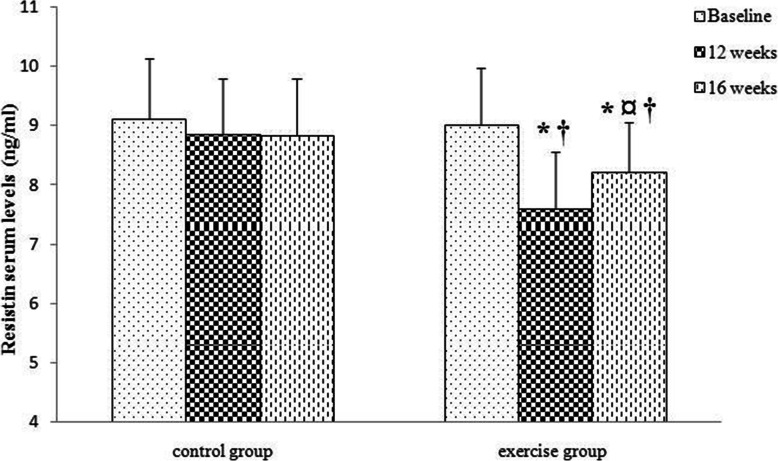
Comparison of serum levels of resistin in the two groups (control and exercise) and three times (baseline, 12 weeks, 16 weeks). Data are reported as Mean ± SEM *: significantly different from baseline,¤: significantly different from 12 weeks, †: significantly different from the control group. p<0.05

The results of statistical analysis also showed that resistin levels decreased significantly after 12 weeks of combined exercise and increased significantly after 4 weeks of detraining. However, resistin levels are still significantly lower in comparison to the baseline. Resistin levels did not change significantly during 16 weeks of study in the control group. There was a significant difference in resistin levels between the control and exercise groups following 12 weeks and 16 weeks (Fig. 7).

**Fig. 8:**
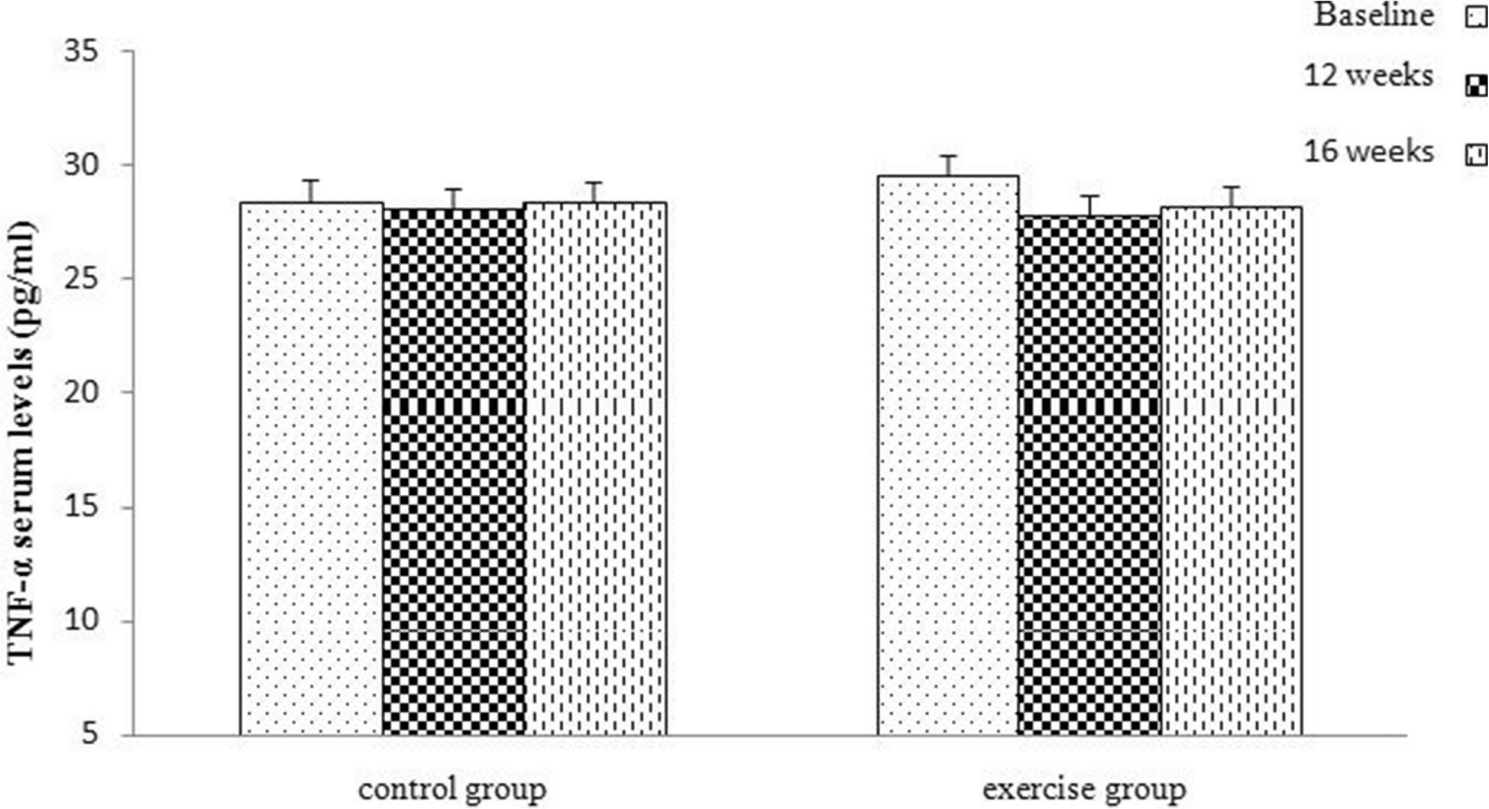
Comparison of serum levels of TNF-α in the two groups (control and exercise) and in three times (baseline, 12 weeks, 16 weeks). Data are reported as Mean ± SEM, TNF-α: tumor necrosis factor-alpha)

The results indicated that TNF-a levels did not change significantly in the exercise and control groups after 12 and 16 weeks (Fig. 8).

## Discussion

In the present study, the signs of puberty (bone age, uterine length, ovarian volume, LH, FSH, and LH/FSH ratio) decreased in both the exercise and control group after 16 weeks from the beginning of the study. However, there are two important points: 1- The rate of reduction of the mentioned factors in the exercise group was faster; 2- the FSH levels, LH/FSH ratio, and ovarian volume did not change significantly in the control group even after 16 weeks showing that exercise combined with medication (in the exercise group) probably has a better effect on preventing precocious puberty in obese and overweight girls. Physical exercise specifically causes suppression of hypothalamic pulsatile release of GnRH, limits pituitary secretion of luteinizing hormone and, to a lesser extent, follicle-stimulating hormone, which, in turn, limits ovarian stimulation [36]. Physical activity may influence aromatase activity by reducing adiposity, which reduces the synthesis of sex hormones or may have effects independent of a change in adiposity, including a reduction in insulin levels, which, in turn, increases sex hormone-binding globulin (SHBG) levels and decreases estradiol bioavailability [37] thus delaying puberty. In the present study, the lipid profile (TC, TG, LDL), %BF and FMI specifically decreased in the exercise group, which could be the reason for a significant reduction of LH and FSH while in the control group, lipid profile did not change significantly. On the other hand, the results of the present study pointed to a significant and direct relationship between change in TG, LDL, weight, BMI and change in LH levels after 12 weeks of combined exercise. Therefore, lowering the lipid profile will be accompanied by reduced LH. Another interesting fact is that insulin resistance in obese subjects is associated with compensatory hyperinsulinemia and decrease the levels of liver sex hormone binding protein, which increases the estrogen levels and promotes breast development [38]. Previous studies have shown that insulin resistance improves after exercise [39]. Therefore, it can be concluded that exercise-induced decrease in insulin resistance is involved in delaying puberty in obese subjects.

The findings of the present study further revealed that combined exercise for 12 weeks increased adiponectin in overweight and obese girls with CPP. As previously mentioned, adiponectin is directly related to weight [8]. In the current study, change in adiponectin levels was also directly correlated with change in weight and BMI. In addition, the weight of the girls in the exercise group decreased by 5.57%. Also, BMI SDS reduced by 0.53 unit in the exercise group after 12 weeks of combined exercise, which could be a possible mechanism for increasing adiponectin. From another point of view, the results of a study by Rendo-Urteaga et al. showed that leptin and obesity seem to play a mediation role in the association between early puberty and inflammatory markers. They recommended weight control to control inflammatory disorders arising in early pubertal [40]. As pointed out by Kiezun et al. [10], adiponectin activates the AMP kinase pathway, which inhibits GnRH activity thereby reducing LH. It has been found that adiponectin is a regulator of puberty onset as it inhibits the secretion of kisspeptin and GnRH in the hypothalamus and the release of Growth Hormone (GH) and LH in the pituitary gland, thereby inhibiting the onset of puberty [41]. Indeed, in GT1–7 cells (hypothalamic GnRH neuron cells) adiponectin not only inhibits GnRH secretion [41] but also suppresses kisspeptin mRNA transcription [42]. Therefore, increasing adiponectin in the present study could be a possible mechanism for reducing LH and signs of puberty.

Another finding of the present study was reduced resistin levels after 12 weeks of combined exercise in the girls with precocious puberty. In line with the findings of the present study, Marcelinoet et al. (2017) confirmed the inverse association between resistin and physical activity in the general adult population and stated that resistin is lower in the people who do more than 20 min of physical activity every day than the people with sedentary lifestyles [15]. Amount of fat mass is undeniably one of the strongest correlates of circulating resistin and change in serum resistin is positively correlated with changes in BMI, body fat and fat mass [43]. Since the results of the present study showed a decrease in weight, BMI, lipid profile, and fat mass, therefore, it can be a possible mechanism for a decrease in circulating levels of resistin in the present study. As in the case of adiponectin, resistin is expressed in the ovarian cells of various species, where it directly affects ovarian steroidogenesis [11]. Resistin increases androgenic action in the ovary [44]. Accordingly, decreased resistin, as observed in this study, may be the possible cause of reduced signs of puberty.

Besides, the present study also evaluated the effects of 4 weeks of detraining. According to the principle of reversibility, training-induced physiological adaptations are transitory and may disappear when the training load is not sufficient. A better understanding of the phenomenon of detraining determines how long the beneficial effects of exercise remain and is essential for designing children’s exercise programs. The findings of the present study revealed that after 4 weeks of detraining, the positive effects of exercise on adiponectin remained. There may be several reasons for this: 1. After 4 weeks of cessation of exercise, the weight and BMI increased, but it was still less than the initial amount before exercise, and since adiponectin is directly related to weight and BMI [8], so this could be a possible mechanism for adiponectin levels remaining high; 2. An interesting finding of Jeon et al. (2013) was that adiponectin extremely increased after 6 weeks of detraining not following 12 weeks of exercise training [45]. In fact, there is a potential effect even after 6 weeks of training; 3. In the present study, 4 weeks of detraining did not thoroughly eliminate the effects of regular combined exercise. As Agarwal et al. (2012) pointed out, 2 weeks of detraining cannot completely eliminate the beneficial effects of regular exercise and further cessation of exercise may result in complete reversal of the beneficial effects [32].

Furthermore, the results of the current study showed that 4 weeks of detraining increases resistin levels, but resistin levels are still significantly lower than basic levels before exercise. This finding demonstrates that 12 weeks of combined exercise was so effective that the positive effects remained after 4 weeks of detraining. Research in this area is limited, however.

It is known that endurance exercise affects the secretion of pro-inflammatory cytokines [16]. Also, the results of a previously published study by the authors showed that aerobic exercise reduced cytokine CRP levels in girls with precocious puberty [46]; Therefore, the authors hypothesized that combined exercise would also reduce TNF-α. But this study yielded a different result: after 12 weeks of combined exercise and 4 weeks of detraining, TNF-α levels did not change significantly in obese girls with precocious puberty. However, the results related to the effects of exercise on TNF-α were very different. In line with the results of this study, Conraads reported that a combination of endurance and resistance exercises did not affect the plasma levels of TNF-α [47]. But Park (2015) examined the effect of combined exercise on postmenopausal women with abdominal obesity and concluded that after 12 weeks of combined exercise, visceral fat and TNF-α reduced [16]. The difference in the results may be due to differences in the age and physical conditions of the participants. In Heidarianpour et al.’s study [46], participants had normal weight, while in the current study, overweight and obese girls were examined. On the other hand, the previous study used aerobic exercise, but the present study used combination exercises with different intensities. Collectively, more research is needed to resolve the existing ambiguities.

### Study strength and limitations

To authors’ knowledge, this is the first study to evaluate the changes in adipokines, cytokine, and signs of pubertal development after exercise training in overweight and obese girls with precocious puberty. The results of this study provide novel insight into the role of exercise as a non-pharmacological effective intervention in modulating precocious puberty as well as the management of childhood obesity. In addition, this study is constrained by several limitations. First of all, the sample size was small. Another limitation was that the follow-up period was short. In addition, the impact of diet and chemicals and industry were not controlled and the changes in participants’ dietary or physical habits were not monitored in this study. Also in this study, leptin and other adipokines related to obesity were not measured. However, the samples participating in the study were matched in terms of disease onset and duration of drug use.

## Conclusion

It can be concluded, based on the findings, that regular combined exercise in combination with GnRH agonist medication is likely to improve the status of adipokines and prevent puberty. Accordingly, the results of the present study support the use of combined exercise in overweight and obese girls with precocious puberty. Furthermore, since the positive effects of exercise fade away during the detraining period, the authors recommend that these children exercise regularly.

## Data Availability

The datasets generated and/or analysed during the current study will be available from the corresponding author on reasonable request.

## Abbreviations

GnRH: Gonadotropin-releasing hormone
LH: Luteinising hormone
FSH: Follicle-stimulating hormone
GnRHa: Gonadotropin-releasing hormone analogues
CPP: Central precocious puberty
MRI: Magnetic resonance imaging
CT: Computerized tomography
BMI: Body mass index
FMI: Fat mass index
BF: Body fat
TNF-α: Tumor necrosis factor-alpha
NF-Κb: Nuclear factor-kappa Beta
CDC: Centers for disease control
SBP: Systolic blood pressure
DBP: Diastolic blood pressure
Vo_2_peak: Peak oxygen uptake
6MWT: 6-min walk test
TC: Total cholesterol
TG: Triglycerides
HDL: High-density lipoprotein
LDL: Low-density lipoprotein
SHBG: Sex hormone-binding globulin
